# Genetic Association between Serum 25-Hydroxyvitamin D Levels and Lung Function in Korean Men and Women: Data from KNHANES 2011–2012

**DOI:** 10.3390/nu10101362

**Published:** 2018-09-23

**Authors:** Clara Yongjoo Park, So-Young Kwak, Garam Jo, Min-Jeong Shin

**Affiliations:** 1 Department of Food and Nutrition, Human Ecology Research Institute, Chonnam National University, Gwangju 61186, Korea; parkcy@jnu.ac.kr; 2 Department of Public Health Sciences, BK21PLUS Program in Embodiment: Health-Society Interaction, Graduate School, Korea University, Seoul 02841, Korea; joilfille@korea.ac.kr (S.-Y.K.); grcho94@korea.ac.kr (G.J.)

**Keywords:** vitamin D, single-nucleotide polymorphism, KNHANES, lung function, FVC, FEV1

## Abstract

The association between single-nucleotide polymorphisms (SNPs) in the vitamin D metabolic pathway and lung function is unknown. We examined the association between five SNPs on *DHCR7*, *GC*, *CYP2R1*, and *CYP24A1* along with serum 25-hydroxyvitamin D (25(OH)D) levels and lung function in older Korean men (*n* = 758) and women (*n* = 837). Lung function was determined by forced vital capacity (FVC) and forced expiratory volume in 1 s (FEV1) from the data in the Korea Nutrition and Health Examination Survey 2011–2012. Genetic risk score (GRS) was calculated by the number of 25(OH)D-decreasing alleles of the five SNPs. Our results showed that increases in GRS were associated with reduced 25(OH)D levels (*p* < 0.05 for both sexes). In the entire population, FVC and FEV1 were associated with both GRS and 25(OH)D levels. In women, FVC and FEV1 were negatively associated with GRS (β-coefficient (95% CI): −0.022 (−0.039, −0.005) and −0.020 (−0.035, −0.005), respectively; both *p* < 0.05), but not with 25(OH)D. However, in men, FVC and FEV1 were positively associated with 25(OH)D (β-coefficient (95% CI): 0.008 (0.001, 0.016) and 0.008 (0.002, 0.015), respectively; both *p* < 0.05), but not with GRS. In conclusion, lung function was associated with genetic variation in Korean women and with 25(OH)D in Korean men.

## 1. Introduction

The relationship between serum 25-hydroxyvitamin D (25(OH)D) concentration (an indicator of vitamin D status) and lung function has been inconsistently reported in various populations. Lung function, assessed by forced vital capacity (FVC) and forced expiratory volume in 1 s (FEV1) measurements, was positively associated with 25(OH)D serum concentration in healthy adults as observed in cross-sectional studies [1,2,3]. In one prospective cohort study, lower 25(OH)D levels were associated with a more rapid decline in lung function and an increased risk of chronic obstructive pulmonary disease (COPD) [4], whereas in another no associations were found between 25(OH)D levels and longitudinal changes in lung function [5]. In rodents, vitamin D-deficient offspring had smaller lung volume, impaired lung function, and increased tracheal contractility compared with vitamin D replete counterparts [6]. However, the benefit of vitamin D supplementation on lung function in humans is not clear. Studies on patients with COPD and/or asthma are inconclusive [7,8,9,10,11,12,13]. On the other hand, vitamin D supplementation in healthy people seemingly benefits current and former smokers, but not those who never smoked [8]. These reports indicate that the benefit of vitamin D on lung function can differ by health status and lifestyle choice. More specific analyses are necessary to clearly understand the underlying mechanism relating 25(OH)D levels and lung function.

Discrepancies in the effect of vitamin D supplementation as observed in randomized controlled trials on lung function may be due to different genetic characteristics of the study populations or interactions between genetics and environment. Previous genome-wide association studies (GWAS) have identified SNPs associated with circulating 25(OH)D levels [14,15]. These include SNPs located on genes *DHCR7*, *GC*, *CYP2R1*, and *CYP24A1*, which are involved in subcutaneous cholesterol synthesis, vitamin D-binding and transport, hepatic hydroxylation of vitamin D, and catabolism of the active vitamin D metabolite, respectively [14]. A recent cross-sectional study using the Framingham Offspring Cohort revealed associations between FEV1 and SNP polymorphisms of rs11819875 (*CYP2R1*) and rs10877013 (*CYP27B1*) that affect genes involved in the conversion of vitamin D to 25(OH)D and then to 1,25-dihydroxy vitamin D (1,25(OH)_2_D), respectively [5]. However, when meta-analyzed in replication cohorts, the association was null [5]. Despite the larger sample size in the meta-analysis, unknown and different characteristics of the populations may interfere with the association between SNP genotype and FEV1. It has been demonstrated that genotype is associated with 25(OH)D status, especially when exposed to vitamin D-increasing environments, such as high vitamin D intake or greater UVB exposure [16,17]. In addition, the association between genotype and lung function in relatively healthy adults may differ by race and sex [18,19,20,21,22], but to date, most vitamin D-related specific genotypes have been studied in Whites with no sex analyses [5]. Therefore, in this study, we assessed the relationship between genetic variants involved in vitamin D metabolism and lung function in relatively healthy older Korean men and women.

## 2. Materials and Methods

### 2.1. Study Participants

Data were collected from the Korea National Health and Nutrition Examination Survey (KNHANES) 2011–2012. KNHANES is a cross-sectional and nationally representative survey conducted by the Korean Centers for Disease Control and Prevention (KCDC). Details of KNHANES are available elsewhere [23]. Among the 16576 subjects who participated in KNHANES 2011–2012 (aged 1 year and older), pulmonary function was assessed in 6633, as spirometry was performed in adults ≥40 years. Participants missing serum 25(OH)D levels (*n* = 236), had cancer or were pregnant/lactating (*n* = 282), or missing covariates (*n* = 160) were excluded. Among the remaining 5955 participants, 1595 participants (758 males and 837 females) had DNA available for analysis. We did not find any difference between the characteristics of those that did not have DNA available (*n* = 4360) and our study population. The study protocol was approved by the institutional review board of Korea University (KU-IRB-16-EX-272-A-1), and all subjects gave their written informed consent for inclusion before they participated in the study. All experiments and methods were performed in accordance with relevant guidelines and regulations.

### 2.2. General Characteristics

KNHANES consists of three components: a health interview, a health examination, and a nutrition survey. Information on age, sex, education level, smoking status, current drinking status, and physical activity was collected during the health interview. Education level was categorized as the completion of elementary school or less and the completion of middle school, high school, and university or higher. Smoking status was divided into three groups: never smoked, former smoker, and current smoker. Current drinking was defined as consuming ≥1 alcoholic drinks/month during the previous year. Participants were determined as physically active if they participated in any of the following activities during the past week: walking for ≥30 min/day for at least 5 days/week, moderate activity for ≥30 min/day for at least 5 days/week, or vigorous activity for ≥20 min/day for at least 3 days/week [24]. Disease outcome variables (hypertension, cardiovascular disease, diabetes, and metabolic syndrome) were defined based on the following criteria: (1) medical history of physician-diagnosis, (2) anthropometric and biochemical measurements, or (3) use of disease-related medications. Anthropometric measurements, including height, weight, and blood pressure, were measured by trained medical staff during the health examination survey. According to standardized protocols, height was measured with a stadiometer (SECA 225, SECA, Hamburg, Germany) to the nearest 0.1 cm, and weight was measured using balance scales (GL-6000-20, G-tech, Republic of Korea) to the nearest 0.1 kg. Body mass index (BMI) was calculated as weight divided by the square of height (kg/m^2^). Waist circumferences were measured to the nearest 0.1 cm at the umbilical level using a measuring tape. Blood pressure was measured three times using a mercury sphygmomanometer (Baumanometer, New York, NY, USA) with subjects in a sitting position. The averages for the second and third measurements were used for calculating systolic blood pressure (SBP) and diastolic blood pressure (DBP).

### 2.3. Biochemical Markers and Serum 25(OH)D Levels

Fasting blood samples were collected to assess biochemical markers. Serum triglyceride (TG), total cholesterol (TC), high-density lipoprotein cholesterol (HDL-C), fasting blood glucose (FBG), aspartate aminotransferase (AST), and alanine aminotransferase (ALT) levels were measured in a central and certified laboratory by enzymatic methods using a Hitachi automatic analyzer 7600 (Hitachi, Tokyo, Japan). Low-density lipoprotein cholesterol (LDL-C) was calculated according to the following equation: LDL-C (mg/dL) = TC (mg/dL)−HDL-C (mg/dL)−TG (mg/dL)/5 for subjects with TG levels <400 mg/dL [25]. Glycated hemoglobin A1c (HbA1c) was measured using high-performance liquid chromatography (HLC-723G7; Tosch, Tokyo, Japan). Serum 25(OH)D levels were measured by radioimmunoassay (DiaSorin, Stillwater, MN, USA). Vitamin D deficiency, insufficiency, and sufficiency were defined as serum 25(OH)D levels being <12 ng/mL, 12–20 ng/mL, and ≥20 ng/mL, respectively [26].

### 2.4. Lung Function Measurements

Lung function was measured using a dry rolling seal spirometer (Vmax series Sensordemics 2130; SensorMedics, Yorba Linda, CA, USA) based on the guidelines of the American Thoracic Society and the European Respiratory Society (ATS/ERS) for standardization [27]. To obtain adequate test results, spirometry was performed at least three times and results were examined to confirm their adherence to ATS/ERS criteria of acceptability, repeatability, and quality control. Detailed spirometry procedures are described elsewhere [28]. FVC and FEV1 measurements were used for analysis.

### 2.5. Genetic Variants of Serum 25(OH)D Levels

We selected five genetic variants for genotyping (*DHCR7* rs12785878, *GC* rs2282679, *CYP2R1* rs10741657 and rs12794714, and *CYP24A1* rs6013897) based on a previous GWAS available at the time of preparation of the manuscript related to vitamin D deficiency (*p* = 2.1 × 10^−27^, 1.9 × 10^−109^, 3.3 × 10^−20^, and 1.8 × 10^−9^, and 6.0 × 10^−10^, respectively) [14] and its use in various Mendelian randomization studies [29,30,31]. These SNPs were reported to be associated with 25(OH)D in recent GWAS performed in Whites [32,33]. The associations and effect size of these genetic variants in Koreans and other races are discussed in detail elsewhere [34].

DNA (300 ng) was extracted from peripheral blood samples and genotyped (DNA Link, Seoul, Korea). According to the manufacturer’s protocols, rs12785878 (*DHCR7*), rs10741657 (*CYP2R1*), rs12794714 (*CYP2R1*), and rs6013897 (*CYP24A1*) were genotyped using the SNPtype assay (Fluidigm, San Francisco, CA, USA), and rs2282679 (*GC*) was genotyped using the TaqMan assay (ABI, Foster City, CA, USA). We calculated the genetic risk score (GRS) of the five genetic variants by adding the number of alleles related to lower serum 25(OH)D levels. Subjects were categorized into GRS quartiles to compare general characteristics among the groups. As no individual had a GRS of 0, the quartile ranges were 1–4, 5, 6, and 7–10.

### 2.6. Statistical Analysis

The characteristics of study participants are expressed as means ± standard error for continuous variables and as percent and frequency for categorical variables. Differences in characteristics according to sex were assessed by Student’s *t*-test. Characteristics were compared among GRS quartiles and among vitamin D groups by general linear models for biochemical markers (after adjusting for sex and age), and the chi-square test for categorical variables such as education, smoking status, current drinking status, and physical activity. Associations between genotypes and serum 25(OH)D levels were tested using an additive linear regression model. Serum TG, FBG, AST, and ALT values were log-transformed to mimic a Gaussian distribution. The effect allele frequencies were calculated using the following formula: (number of heterozygotes + 2 × number of homozygotes of the effect allele)/(2 × total number of subjects). Multiple linear regression models were used to examine the individual and combined effects of GRS and serum 25(OH)D levels on lung function after adjusting for age, sex, BMI, education level, smoking status, current drinking status, and physical activity. Except for the comparison of characteristics among GRS quartiles, the GRS was used as a continuous variable in the analyses. Results are reported as beta coefficients and at 95% confidence interval (CI). In addition, the adjusted R-square values are presented to compare each model. Because of the substantial differences in lung function between men and women, all analyses were stratified based on sex. All statistical analyses were performed using the Stata MP 13 software (Stata Corp. LP, Texas, TX, USA). A two-tailed *p*-value of <0.05 was considered as statistically significant.

## 3. Results

A total of 1595 subjects were included in this study. The frequency of 25(OH)D-decreasing alleles (effect alleles) was similar to that observed in our previous study (Table 1, [34]). Serum 25(OH)D level was associated with the frequency of effect alleles in rs12785878, rs2282679, rs10741657, and rs12794714, but not with that of rs6013897 (Table 1). The distribution of 25(OH)D values differed between sex and the mean 25(OH)D was higher in men compared to that in women (Supplementary Table S1). Age, physical activity, BMI, and the genotype distribution did not differ between men and women, except for rs6013897.

Vitamin D status decreased with higher GRS in the population (Supplementary Table S2). Mean FVC and FEV1 differed between GRS quartiles, where Q2 had the highest and Q4 had the lowest mean values. There were no differences between GRS quartiles in terms of demographics, lifestyle variables, and biochemical markers. Mean SBP was higher than normal values (<120 mmHg) but mean DBP was within the normal range (<80 mmHg) [35]. Mean FBG and HbA1c were higher than normal levels (<100 mg/dL and <5.7%, respectively) [36]. When analyzed based on sex, GRS was negatively associated with 25(OH)D, but not with lung function, demographics, lifestyle variables, or biochemical markers in men (Table 2). However, in women, serum 25(OH)D levels, FVC, FEV1, and age differed between GRS quartiles.

When subjects were grouped by 25(OH)D status, only 30% had 25(OH)D ≥20 ng/mL, the target 25(OH)D status for Adequate Intake by the Korean Dietary Reference Intakes [37] (Supplementary Table S3). In the study population, mean FVC and FEV1 increased, and the proportion of men with higher 25(OH)D levels increased. Among subjects with higher 25(OH)D levels, the proportions of ever-smokers (former and current) and current drinkers were higher, possibly due to a higher proportion of men. Mean age increased with higher 25(OH)D in the whole population and in both men and women when analyzed by sex. In men, FVC, FEV1, age, education, physical activity, TG, and HDL-C differed according to 25(OH)D levels (Table 3). When women were separately analyzed, their lung function did not differ according to 25(OH)D status. 

Both GRS and 25(OH)D levels were independently associated with FVC and FEV1 in the entire population (Table 4). When GRS and 25(OH)D level were both included in the regression model, FEV1 was associated with both GRS and 25(OH)D level. In this combined model, 25(OH)D status was positively associated with FVC; moreover, a trend of association between FVC and GRS was observed (*p* = 0.07). When the association with lung function was determined by sex, GRS was negatively associated with lung function in women, whereas serum 25(OH)D was positively associated with FVC and FEV1 in men. This relationship did not change when both GRS and 25(OH)D were included in the regression model.

## 4. Discussion

In this study, we found a genetic association between SNPs involved in the vitamin D metabolic pathway and lung function in older Korean men and women. Specifically, in women, SNPs strongly associated with lower 25(OH)D levels were negatively associated with FVC and FEV1. However, no associations between serum 25(OH)D levels and these lung function parameters were detected in women. On the other hand, in men, serum 25(OH)D levels significantly contributed to FVC and FEV1, but genetic associations were not evident. The clinical relevance of the associations due to the small beta coefficients must be determined.

Several previous studies have reported an association between serum 25(OH)D levels influencing genetic variants and lung function or relevant respiratory diseases [5,38,39,40,41,42], but analyses of genetic associations performed according to sex are limited. It was previously reported that genotypes of rs10877013 (*CYP27B1*) and rs11819875 (*CYP2R1*) were associated with a change in FEV1 in the Framingham cohort (*n* = 3230), but not in a meta-analyzed replication cohort (*n* = 7246) [5]. Following a Mendelian randomization of genotypes of four SNPs, each located on *DHCR7*, *GC*, *CYP2R1*, and *CYP24A1* (including rs2282679 (*GC*)), no relationship was observed between genotype and asthma [38]. However, a meta-analysis including Whites and Asians revealed a different risk for COPD according to *GC* SNP variants [39]. Some SNPs on *VDR*, *GC*, *CYP2R1*, *CYP24A1*, and *CYP27B1* were associated with higher risk of non-small cell lung cancer (NSCLC) in Chinese people [40,41], and some SNPs on *CYP2R1* and *CYP27B1* were associated with the rate of FEV1 change over a ten-year period [42]. One small study in Korean COPD patients, predominantly consisting of men, found that genotype was associated with vitamin D deficiency (25(OH)D < 20 ng/mL), but vitamin D deficiency was associated with lower FEV1/FVC, regardless of *GC* genotype. The genotypes of specific variants of SNPs rs4588 and rs7041 [43] were assessed in this study. These two SNPs are in strong linkage disequilibrium with rs2282679 assessed in our study and the results are similar to our results observed in men. Another study in male COPD patients and healthy controls found an interaction between smoking and *GC* polymorphism on FEV1 [44]. No studies in Koreans were performed in cohorts with adequate samples of women. Further research on the associations of vitamin D metabolism-related SNPs and lung function in Korean women is required.

In our study, we observed that increases in the number of serum vitamin D-decreasing alleles are negatively associated with FEV1 and FVC in women but not in men. We speculate that women may be more sensitive to genetic regulation of lung function. A report in Australian twins showed a sex difference in lung function heritability, where females are more strongly affected by genetics [19]. In Swedish twins, the genes related to FEV1 were identical in men and women, but the influence of genes was approximately 4-fold greater in women compared to men when adjusted by smoking and respiratory symptoms [18]. In another Swedish twin cohort, no sex difference in heritability of lung function was observed; however, opposite-sex twins were not included in the study [20]. Our study shows that lung function in Korean women may also be more strongly influenced by vitamin D-related genetic factors compared with that in men.

On the other hand, the positive association between the serum 25(OH)D levels and lung function was found to be greater than the genotype effects in men, which is in line with previous observations in healthy adults. In Whites, the rate of change in FEV1 following increasing 25(OH)D was greatest when 25(OH)D levels were <12 ng/mL, attenuated between 12 and 40 ng/mL, and plateaued around 40 ng/mL [5]. Additionally, 25(OH)D status was positively associated with FEV1 and FVC in Koreans, where lung function rapidly increased with 25(OH)D at low levels (5–10 ng/mL) and tended to plateau near 20–25 ng/mL [2]. Approximately 64% of men and 76% of women in our study had 25(OH)D levels ≤20 ng/mL. These low 25(OH)D values may have been advantageous in detecting a positive correlation between 25(OH)D levels and lung function in our population. However, despite the higher fraction of women with 25(OH)D levels <12 ng/mL (20% in women vs. 9% in men), 25(OH)D status was associated with lung function only in men.

It can be speculated that sex hormones may be involved in the different association of 25(OH)D and lung function between men and women. Sex steroids are associated with lung development even before the neonatal period; lung volume is greater in boys than in girls, and conducting airways are larger in men compared to women [45]. Likewise, testosterone may be associated with 25(OH)D levels and lung function in men. Men with COPD were reported to have low testosterone, and reduced testosterone is associated with lower lung function [46,47,48]. This is further supported by the results that vitamin D supplementation increased testosterone levels in men [49]. In elderly Dutch men, 25(OH)D levels were positively associated with total and bioavailable testosterone, regardless of the genotypes of rs12785878, rs10741657, or several other SNPs in the vitamin D pathway [50]. On the other hand, whether vitamin D supplementation increases sex-hormone levels and lung function in older women is unknown. Estrogen receptors in the lungs respond to circulating estrogen and may be associated with NSCLC [51], and the lung diffusing capacity is affected by the menstrual cycle [52]. However, the potential effect of 25(OH)D levels on sex organs to produce gonadal hormones, which may possibly affect lung function, in women in our study may be weak because approximately 70% were post-menopausal. Therefore, it is possible that the genotype-independent association between serum 25(OH)D levels and lung function observed in the Korean men in our study occurred through increasing testosterone levels, which was not experimentally proven in the present study. Further research is required to understand the specific mechanism related to this association.

Our results indicate an independent contribution of serum 25(OH)D and genetic variation of vitamin D-related genes on lung function. The association between lung function and GRS was not affected by 25(OH)D levels, and the association between lung function and 25(OH)D levels were not affected by GRS. In line with our results, in a cohort of Korean COPD patients primarily consisting of males (97.7%), low 25(OH)D was associated with FEV1/FVC [43] regardless of rs4081 or rs7041 genotypes (both located on *GC*). Coronary lesions [53] and tuberculosis [54] have also been associated with 25(OH)D levels, but not with vitamin D relevant SNPs. On the other hand, in Korean men, emphysema was associated with the genotypes of rs4081 and rs7041, but not with vitamin D deficiency [43]. Polymorphisms of rs4081 and rs7041 result in changes in amino acids and the function of the vitamin D-binding protein [55]. The vitamin D-binding protein binds to and transports vitamin D metabolites but is also thought to affect innate immunity independent of vitamin D transport [55,56]. Variation of SNPs on *VDR* and *CYP27A1* was associated with lethal prostate cancer even after adjustment for 25(OH)D levels [57]. These reports indicate that SNPs of genes play a role in the vitamin D metabolic pathway, and thus proteins in the vitamin D metabolic pathway may have physiologic roles independent of the 25(OH)D levels.

This study, however, has some limitations. Due to the design of KNHANES, gene analysis is not subject to the stratified multi-stage probability sampling. Therefore, the results should not be interpreted as nationally representative of Koreans. Nonetheless, mean FVC, FEV1, 25(OH)D and other characteristics of our study population are similar to the general Korean population [2]. Second, the serum collection season was not adjusted for, as the date of collection was not available in the public database. However, it is very unlikely that those with particular genotypes or lung function were randomly sampled in a particular season. The association between genetic variation and 25(OH)D levels, as reported in previous studies [42,58,59], implies that seasonal interference on the association between lung function and GRS or 25(OH)D levels may be small. The association between 25(OH)D and lung function have been reported in many populations regardless of adjustment for seasonality [1,2,4]. Another limitation is the cross-sectional design of this study. A causal relationship between 25(OH)D and lung function in Korean men must be determined in a randomized controlled trial. In addition, the mechanism of genetic effects on lung function in women needs further investigation.

To our knowledge, this is the first study to report associations between lung function and 25(OH)D levels in Korean men and women. We also found an independent relationship between the genetic variation of vitamin D-related genes and 25(OH)D levels with lung function albeit the strong association between genotype and 25(OH)D in Koreans. Additionally, lung function may be maximized by increasing 25(OH)D levels regardless of the genotype of some SNPs related to vitamin D metabolism in Korean men.

## Figures and Tables

**Table 1:** Association between five single-nucleotide polymorphisms (SNPs) and 25(OH)D status.

SNP	Nearest Gene	Chromosome	Serum 25(OH)D-Decreasing Allele	Effect Allele Frequency	Beta-Coefficient ^1^	*p*-Value ^1^
rs12785878	*DHCR7*	11	G	0.617	−0.009	0.003
rs2282679	*GC*	4	G	0.304	−0.008	0.004
rs10741657	*CYP2R1*	11	G	0.610	−0.007	0.016
rs12794714	*CYP2R1*	11	A	0.394	−0.009	0.003
rs6013897	*CYP24A1*	20	T	0.886	−0.002	0.374

25(OH)D, 25-hydroxyvitamin D; *DHCR7*, 7-dehydrocholesterol reductase; *GC*, vitamin D-binding protein; *CYP2R1*, cytochrome P450 family 2 subfamily R member 1, *CYP24A1*, cytochrome P450 family 24 subfamily A member 1 ^1^ Beta coefficients and *p*-values were obtained from linear regression between serum 25(OH)D levels and genotype.

**Table 2:** Characteristics of study participants according to genetic risk score quartile and sex.

	Males (*n* = 758)					Females (*n* = 837)				
	Genetic Risk Score (5 SNP; 1–10)					Genetic Risk Score (5 SNP; 1–10)				
	1–4 (*n* = 174)	5 (*n* = 172)	6 (*n* = 188)	7–10 (*n* = 224)	*p*-Value ^1^	1–4 (*n* = 210)	5 (*n* = 162)	6 (*n* = 215)	7–10 (*n* = 250)	*p*-Value ^1^
Mean serum 25(OH)D levels (ng/mL)	19.1 ± 0.5	19.3 ± 0.4	18.1 ± 0.3	18.0 ± 0.4	0.013	17.6 ± 0.4	17.1 ± 0.5	16.6 ± 0.4	15.6 ± 0.3	0.001
FVC (L)	4.13 ± 0.05	4.15 ± 0.05	4.16 ± 0.05	4.10 ± 0.05	0.472	2.97 ± 0.03	2.89 ± 0.04	2.96 ± 0.03	2.90 ± 0.03	0.008
FEV1 (L)	3.09 ± 0.05	3.14 ± 0.05	3.14 ± 0.04	3.07 ± 0.04	0.452	2.37 ± 0.03	2.29 ± 0.03	2.36 ± 0.03	2.31 ± 0.03	0.004
Age (years)	56.8 ± 0.8	57.2 ± 0.8	56.7 ± 0.7	56.7 ± 0.7	0.762	57.3 ± 0.7	57.2 ± 0.8	55.3 ± 0.7	55.9 ± 0.6	0.045
Body mass index (kg/m^2^)	24.0 ± 0.2	24.8 ± 0.2	24.7 ± 0.2	24.7 ± 0.2	0.009	24.4 ± 0.2	24.7 ± 0.2	23.9 ± 0.2	24.5 ± 0.2	0.069
Education (elementary school/middle school/high school/university) (%)	16.8/18.5/38.7/26.0	21.8/21.2/28.8/28.2	22.2/17.8/34.1/26.0	18.9/18.0/30.6/32.4	0.586	39.7/18.7/30.1/11.5	38.3/14.8/26.5/20.4	35.1/15.0/32.7/17.3	37.8/14.2/30.5/17.5	0.498
Smoking status (never/former/current smoker) (%)	16.8/49.1/34.1	16.5/48.8/34.7	11.9/51.9/36.2	14.9/44.6/40.5	0.627	93.8/2.9/3.4	93.8/2.5/3.7	91.1/3.7/5.1	91.1/3.7/5.3	0.897
Current drinker (%, N)	75.6 (130)	66.5 (113)	76.2 (141)	75.7 (168)	0.114	37.3 (78)	37.0 (60)	34.6 (74)	34.6 (85)	0.891
Physical activity (%, N)	45.1 (78)	45.3 (77)	41.6 (77)	50.2 (111)	0.377	44.5 (93)	37.7 (61)	37.9 (81)	44.3 (109)	0.292
SBP (mmHg)	124.1 ± 1.1	125.1 ± 1.2	124.6 ± 1.2	125.2 ± 1.1	0.521	120.5 ± 1.2	123.6 ± 1.3	120.9 ± 1.2	122.0 ± 1.1	0.284
DBP (mmHg)	81.0 ± 0.8	79.7 ± 0.8	80.9 ± 0.8	80.2 ± 0.7	0.631	75.3 ± 0.7	76.4 ± 0.7	76.0 ± 0.6	76.2 ± 0.7	0.205
TG (mg/dL) ^2^	167.4 ± 10.4	161.8 ± 6.9	178.6 ± 11.5	171.0 ± 9.3	0.595	130.6 ± 5.4	129.4 ± 5.5	130.6 ± 5.5	137.1 ± 10.1	0.459
TC (mg/dL)	192.3 ± 2.6	187.9 ± 2.6	188.1 ± 2.6	196.6 ± 2.5	0.201	196.0 ± 2.5	197.7 ± 2.6	198.7 ± 2.6	197.5 ± 2.3	0.924
HDL-C (mg/dL)	47.9 ± 0.9	44.8 ± 0.8	45.1 ± 0.8	46.7 ± 0.8	0.445	50.3 ± 0.9	50.7 ± 0.9	50.6 ± 0.8	50.8 ± 0.8	0.590
LDL-C (mg/dL)	113.4 ± 2.4	111.3 ± 2.5	110.3 ± 2.4	118.1 ± 2.4	0.166	119.4 ± 2.2	121.5 ± 2.5	122.3 ± 2.3	120.9 ± 2.0	0.872
FBG (mg/dL) ^2^	103.1 ± 1.7	105.4 ± 1.8	107.4 ± 1.8	103.0 ± 1.3	0.782	101.6 ± 1.8	100.1 ± 1.1	98.0 ± 1.3	99.8 ± 1.4	0.560
HbA1c (%) ^2^	5.89 ± 0.06	5.93 ± 0.06	6.02 ± 0.08	5.81 ± 0.04	0.438	5.99 ± 0.07	5.87 ± 0.05	5.85 ± 0.05	5.83 ± 0.05	0.295
AST (IU/L) ^2^	25.4 ± 0.9	26.9 ± 1.0	25.6 ± 0.8	26.1 ± 1.8	0.576	21.9 ± 0.5	22.2 ± 0.8	22.1 ±0.7	24.5 ± 2.5	0.092
ALT (IU/L) ^2^	26.6 ± 1.5	28.4 ± 1.5	25.0 ± 0.9	27.5 ± 3.5	0.228	20.0 ± 0.8	20.5 ± 1.1	20.6 ± 1.4	19.9 ± 1.2	0.488

FVC, forced vital capacity; FEV1, forced expiratory volume in 1 s; SBP, systolic blood pressure; DBP, diastolic blood pressure; TC, total cholesterol; TG, triglycerides; HDL-C, high-density lipoprotein cholesterol; LDL-C, low-density lipoprotein cholesterol; FBG, fasting blood glucose; HbA1c, glycated hemoglobin A1c; AST, aspartate aminotransferase; ALT, alanine aminotransferase. Values were expressed as means ± standard error for continuous variables and number of counts and percentage for categorical variables. ^1^ Statistical difference among genetic risk score were determined using a general linear model for continuous variables after adjusting for age and chi-squared test for categorical variables. ^2^ Tested after log-transformation.

**Table 3:** Characteristics of study participants based on serum 25-hydroxyvitamin D (25(OH)D) categories and sex.

	Males (*n* = 758)				Females (*n* = 837)			
	Serum 25(OH)D Categories				Serum 25(OH)D Categories			
	0–12 ng/mL (*n* = 68)	12–20 ng/mL (*n* = 416)	≥20 ng/mL (*n* = 274)	*p*-Value ^1^	0–12 ng/mL (*n* = 169)	12–20 ng/mL (*n* = 464)	≥20 ng/mL (*n* = 204)	*p*-Value ^1^
Mean serum 25(OH)D levels (ng/mL)	10.6 ± 0.1	16.0 ± 0.1	24.4 ± 0.3	<0.001	10.0 ± 0.1	15.6 ± 0.1	24.8 ± 0.3	<0.001
FVC (L)	4.12 ± 0.09	4.13 ± 0.03	4.14 ± 0.04	0.007	2.99 ± 0.04	2.95 ± 0.02	2.85 ± 0.04	0.231
FEV1 (L)	3.04 ± 0.09	3.13 ± 0.03	3.08 ± 0.04	0.006	2.39 ± 0.04	2.36 ± 0.02	2.23 ± 0.03	0.312
Age (years)	55.4 ± 1.4	56.4 ± 0.5	57.8 ± 0.6	0.031	54.6 ± 0.7	55.2 ± 0.4	60.4 ± 0.7	<0.001
Body mass index (kg/m^2^)	24.7 ± 0.3	24.6 ± 0.1	24.4 ± 0.2	0.407	24.5 ± 0.3	24.4 ± 0.1	24.2 ± 0.2	0.530
Education (elementary school/middle school/high school/university) (%)	9.1/24.2/34.9/31.8	18.9/16.3/33.3/31.6	23.9/21.3/32.0/22.8	0.024	27.7/21.1/30.1/21.1	35.3/14.1/32.9/17.8	51.2/14.8/24.1/9.9	<0.001
Smoking status (never/former/current smoker) (%)	16.7/37.9/45.5	16.0/46.6/37.4	12.9/53.7/33.5	0.147	91.6/3.6/4.8	90.9/3.5/5.6	96.1/2.5/1.5	0.168
Current drinker (%, N)	69.7 (46)	73.2 (301)	75.4 (205)	0.612	28.9 (48)	40.3 (186)	31.0 (63)	0.009
Physical activity (%, N)	50.0 (33)	41.4 (170)	51.5 (140)	0.027	38.0 (63)	42.9 (198)	40.9 (83)	0.538
SBP (mmHg)	126.6 ± 2.0	124.0 ± 0.8	125.5 ± 1.0	0.848	123.2 ± 1.5	120.9 ± 0.8	122.2 ± 1.2	0.004
DBP (mmHg)	81.1 ± 1.4	80.3 ± 0.5	80.4 ± 0.6	0.736	77.0 ± 0.8	76.3 ± 0.5	74.3 ± 0.6	0.015
TG (mg/dL) ^2^	210.1 ± 19.5	176.3 ± 7.5	150.4 ± 5.1	0.003	135.9 ± 6.6	131.1 ± 5.9	132.1 ± 5.5	0.351
TC (mg/dL)	191.9 ± 5.1	193.2 ± 1.8	189.0 ± 2.0	0.398	196.3 ± 2.8	197.8 ± 1.7	197.6 ± 2.6	0.902
HDL-C (mg/dL)	42.5 ± 1.5	46.1 ± 0.6	47.1 ± 0.6	0.009	50.0 ± 1.0	50.8 ± 0.5	50.6 ± 0.8	0.272
LDL-C (mg/dL)	111.2 ± 4.6	114.9 ± 1.7	112.2 ± 1.9	0.832	120.2 ± 2.3	121.5 ± 1.5	120.5 ± 2.5	0.858
FBG (mg/dL) ^2^	103.5 ± 2.2	105.2 ± 1.2	104.1 ± 1.2	0.841	98.2 ± 1.4	101.1 ± 1.1	98.4 ± 1.0	0.636
HbA1c (%) ^2^	5.91 ± 0.10	5.90 ± 0.04	5.91 ± 0.05	0.750	5.80 ± 0.05	5.91 ± 0.04	5.89 ± 0.05	0.567
AST (IU/L) ^2^	30.6 ± 5.8	25.3 ± 0.5	25.9 ± 0.7	0.883	25.5 ± 3.7	22.1 ± 0.5	22.1 ± 0.5	0.587
ALT (IU/L) ^2^	35.4 ± 11.1	26.6 ± 0.9	25.3 ± 1.0	0.762	21.1 ± 2.0	20.4 ± 0.7	19.1 ± 0.6	0.823

FVC, forced vital capacity; FEV1, forced expiratory volume in 1 s; SBP, systolic blood pressure; DBP, diastolic blood pressure; TC, total cholesterol; TG, triglycerides; HDL-C, high-density lipoprotein cholesterol; LDL-C, low-density lipoprotein cholesterol; FBG, fasting blood glucose; HbA1c, glycated hemoglobin A1c; AST, aspartate aminotransferase; ALT, alanine aminotransferase. Values were expressed as means ± standard error for continuous variables and number of counts and percentage for categorical variables. ^1^ Statistical differences among vitamin D levels were determined using a general linear model for continuous variables after adjusting for age and chi-squared test for categorical variables. ^2^ Tested after log-transformation.

**Table 4:** Individual and combined effect of genetic risk score (GRS) and serum 25-hydroxyvitamin D (25(OH)D) on pulmonary function based on sex in KNHANES data.

	Individual						Combined				
	Adj *R*^2^	GRS		Adj *R*^2^	25(OH)D		Adj *R*^2^	GRS		25(OH)D	
		β-Coefficient (95% CI)	*p*-Value		β-Coefficient (95% CI)	*p*-Value		β-Coefficient (95% CI)	*p*-Value	β-Coefficient (95% CI)	*p*-Value
Total											
FVC (L)	0.648	−0.016 (−0.032, −0.001)	0.036	0.649	0.006 (0.002, 0.010)	0.008	0.649	−0.014 (−0.029, 0.001)	0.070	0.006 (0.001, 0.010)	0.014
FEV1 (L)	0.591	−0.016 (−0.029, −0.003)	0.014	0.592	0.006 (0.002, 0.010)	0.003	0.593	−0.014 (−0.027, −0.001)	0.033	0.005 (0.001, 0.009)	0.006
Males											
FVC (L)	0.256	−0.008 (−0.034, 0.018)	0.545	0.260	0.008 (0.001, 0.016)	0.034	0.259	−0.005 (−0.031, 0.021)	0.686	0.008 (0.000, 0.016)	0.038
FEV1 (L)	0.402	−0.009 (−0.031, 0.013)	0.435	0.406	0.008 (0.002, 0.015)	0.016	0.405	−0.006 (−0.028, 0.016)	0.580	0.008 (0.001, 0.014)	0.019
Females											
FVC (L)	0.315	−0.022 (−0.039, −0.005)	0.013	0.310	0.002 (−0.003, 0.007)	0.360	0.314	−0.002 (−0.038, −0.004)	0.017	0.002 (−0.003, 0.007)	0.534
FEV1 (L)	0.388	−0.020 (−0.035, −0.005)	0.007	0.383	0.002 (−0.003, 0.006)	0.482	0.387	−0.020 (−0.034, −0.005)	0.009	0.001 (−0.003, 0.005)	0.701

Adj *R*^2^, Adjusted R-square; FVC, forced vital capacity; FEV1, forced expiratory volume in 1 s; CI, confidence interval. Beta coefficients of regression models were adjusted for age, sex, BMI, education level, smoking status, current drinking status, and physical activity.

## Supplementary Materials

The following are available online at http://www.mdpi.com/2072-6643/10/10/1362/s1, Table S1: Characteristics of study participants, Table S2: Characteristics of study participants according to genetic risk score (*n* = 1595), Table S3: Characteristics of study participants according to serum 25-hydroxyvitamin D (25(OH)D) categories (*n* = 1595).
