# Supplementary material for: Genetic Association between Serum 25-Hydroxyvitamin D Levels and Lung Function in Korean Men and Women: Data from KNHANES 2011–2012
Source: Nutrients. 2018 Sep 23;10(10):1362. doi: 10.3390/nu10101362 (PMC6212955; doi:10.3390/nu10101362)
Supplement: Supplementary file 1 [file nutrients-10-01362-s001.pdf]

Table S1. Characteristics of study participants.

	All (*n* =1595)	Males (*n* = 758)	Females (*n* = 837)	*p*-value[1]
Mean serum 25(OH)D levels (ng/mL)	17.6 ± 0.1	18.6 ± 0.2	16.7 ± 0.2	<0.001
0–12 ng/mL	237 (14.9)	68 (9.0)	169 (20.2)	
12–20 ng/mL	880 (55.2)	416 (54.9)	464 (55.4)	<0.001
≥20 ng/mL	478 (30.0)	274 (36.2)	204 (24.4)	
Lifestyle and socio-economic factor				
Age (years)	56.6 ± 0.3	56.8 ± 0.4	56.3 ± 0.3	0.343
Education (elementary school/middle school/high school/university) (%)	29.2/17.1/31.5/22.1	19.9/18.8/32.9/28.4	37.7/15.6/30.2/16.5	<0.001
Smoking status (never/former/current smokers) (%)	55.6/24.7/19.7	14.9/48.4/36.7	92.3/3.3/4.5	<0.001
Current drinker (%, N)	53.7 (849)	73.7 (552)	35.7 (297)	<0.001
Physical activity (%, N)	43.5 (687)	45.8 (343)	41.4 (344)	0.078
Disease				
Hypertension (%, N)	42.0 (663)	46.6 (349)	37.8 (314)	<0.001
Cardiovascular disease (%, N)	5.1 (80)	6.1 (46)	4.1 (34)	0.063
Diabetes (%, N)	13.3 (210)	15.8 (118)	11.0 (92)	0.006
Metabolic syndrome (%, N)	43.4 (690)	41.7 (315)	44.9 (375)	0.192
Cardiovascular risk factor				
Systolic blood pressure (mmHg)	123.2 ± 0.4	124.8 ± 0.6	121.7 ± 0.6	<0.001
Diastolic blood pressure (mmHg)	78.1 ± 0.3	80.4 ± 0.4	76.0 ± 0.3	<0.001
Body mass index (kg/m^2)	24.5 ± 0.1	24.5 ± 0.1	24.4 ± 0.1	0.211
Waist circumference (cm)	82.8 ± 0.2	86.5 ± 0.3	81.4 ± 0.3	<0.001
Triglycerides (mg/dL) [2]	150.2 ± 3.1	170.0 ± 4.9	132.3 ± 3.8	<0.001
Total cholesterol (mg/dL)	194.7 ± 0.9	191.6 ± 1.3	197.5 ± 1.2	0.001
HDL cholesterol (mg/dL)	48.5 ± 0.3	46.1 ± 0.4	50.6 ± 0.4	<0.001
LDL cholesterol (mg/dL)	117.5 ± 0.8	113.5 ± 1.2	121.0 ± 1.1	<0.001
Fasting blood glucose (mg/dL) [2]	102.1 ± 0.5	104.7 ± 0.8	99.9 ± 0.7	<0.001
Glycated hemoglobin A1c (%) [2]	5.89 ± 0.02	5.91 ± 0.03	5.88 ± 0.03	0.571
Aspartate aminotransferase (AST, IU/L) [2]	24.3 ± 0.5	26.0 ± 0.7	22.8 ± 0.8	<0.001
Alanine aminotransferase (ALT, IU/L) [2]	23.4 ± 0.6	26.9 ± 1.2	20.2 ± 0.6	<0.001
Genotype distribution				
rs12785878 in *DHCR7* (GG/GT/TT, %)	38.2/47.0/14.8	38.7/46.7/14.6	37.9/47.2/14.9	0.948
rs2282679 in *GC* (TT/TG/GG, %)	48.4/42.5/9.2	49.9/42.0/8.2	47.1/42.9/10.0	0.329
rs10741657 in *CYP2R1* (GG/GA/AA, %)	37.3/47.4/15.3	36.4/48.2/15.4	38.1/46.7/15.2	0.779
rs12794714 in *CYP2R1* (GG/GA/AA, %)	35.7/49.8/14.6	37.1/48.2/14.8	34.4/51.3/14.3	0.447
rs6013897 in *CYP24A1* (TT/TA/AA, %)	78.5/20.2/1.2	80.9/18.3/0.8	76.3/21.9/1.8	0.039

Values are represented as means ± standard error for continuous variables and number of counts and percentage for categorical variables. [1] *p*-values are from student's t-test for continuous variables and chi-squared test for categorical variables assessing the difference between males and females. [2] Tested after log-transformation.

Table S2. Characteristics of study participants according to genetic risk score (*n* = 1595).

	Genetic risk score (5 SNP; 1–10)				
	1–4 (*n* = 384)	5 (*n* = 334)	6 (*n* = 403)	7–10 (*n* = 474)	*p*-value[1]
Mean serum 25(OH)D levels (ng/mL)	18.3 ± 0.3	18.3 ± 0.3	17.3 ± 0.3	16.7 ± 0.2	<0.001
FVC (L)	3.50 ± 0.04	3.54 ± 0.05	3.52 ± 0.04	3.47 ± 0.04	0.027
FEV1 (L)	2.70 ± 0.03	2.73 ± 0.04	2.72 ± 0.03	2.67 ± 0.03	0.014
Age (years)	57.1 ± 0.5	57.2 ± 0.6	56.0 ± 0.5	56.3 ± 0.5	0.100
Males (%, N)	45.3 (174)	51.5 (172)	46.7 (188)	47.3 (224)	0.391
Body mass index (kg/m^2)	24.2 ± 0.2	24.8 ± 0.2	24.3 ± 0.1	24.6 ± 0.1	0.274
Education (elementary school/middle school/high school/university) (%)	29.3/18.6 /34.0/18.1	29.8/18.1 /27.7/24.4	29.1/16.3 /33.3/21.3	28.9/16.0 /30.6/24.6	0.431
Smoking status (never/former/current smoker)(%)	58.9/23.8/17.3	54.2/26.2/19.6	54.4/26.1/19.6	54.9/23.1/22.0	0.591
Current drinker (%, N)	54.6 (208)	52.1 (173)	53.9 (215)	54.1 (253)	0.921
Physical activity (%, N)	44.8 (171)	41.6 (138)	39.6 (158)	47.1 (220)	0.127
SBP (mmHg)	122.2 ± 0.8	124.4 ± 0.9	122.7 ± 0.9	123.5 ± 0.8	0.214
DBP (mmHg)	77.9 ± 0.6	78.1 ± 0.5	78.3 ± 0.5	78.1 ± 0.5	0.962
TG (mg/dL) [2]	147.3 ± 5.6	146.1 ± 4.5	153.0 ± 6.2	153.1 ± 6.9	0.895
TC (mg/dL)	194.4 ± 1.8	192.7 ± 1.9	193.7 ± 1.8	197.1 ± 1.7	0.255
HDL-C (mg/dL)	49.2 ± 0.6	47.7 ± 0.6	48.0 ± 0.6	48.8 ± 0.6	0.762
LDL-C (mg/dL)	116.7 ± 1.6	116.4 ± 1.8	116.9 ± 1.7	119.6 ± 1.5	0.199
FBG (mg/dL) [2]	102.3 ± 1.2	102.8 ± 1.1	102.4 ± 1.1	101.3 ± 1.0	0.564
HbA1c (%) [2]	5.94 ± 0.05	5.90 ± 0.04	5.93 ± 0.05	5.82 ± 0.03	0.074
AST (IU/L) [2]	23.5 ± 0.5	24.6 ± 0.7	23.7 ± 0.5	25.2 ± 1.6	0.989
ALT (IU/L) [2]	23.0 ± 0.8	24.6 ± 1.0	22.6 ± 0.9	23.5 ± 1.8	0.141

FVC, forced vital capacity; FEV1, forced expiratory volume in 1 second; SBP, systolic blood pressure; DBP, diastolic blood pressure; TC, total cholesterol; TG, triglycerides; HDL-C, high-density lipoprotein cholesterol; LDL-C, low-density lipoprotein cholesterol; FBG, fasting blood glucose; HbA1c, glycated hemoglobin A1c; AST, aspartate aminotransferase; ALT, alanine aminotransferase. Values were expressed as means ± standard error for continuous variables and number of counts and percentage for categorical variables. [1] Statistical differences among genetic risk score were determined using general linear model for continuous variables after adjusting for age and sex and chi-squared test for categorical variables. [2] Tested after log-transformation.

Table S3. Characteristics of study participants according to serum 25-hydroxyvitamin D (25(OH)D) categories (*n* = 1595).

	Serum 25(OH)D categories			
	0–12 ng/mL (*n* = 237)	12–20 ng/mL (*n* = 880)	≥20 ng/mL (*n* = 478)	*p*-value[1]
Mean serum 25(OH)D levels (ng/mL)	10.1 ± 0.1	15.8 ± 0.1	24.6 ± 0.2	<0.001
FVC (L)	3.32 ± 0.05	3.51 ± 0.03	3.59 ± 0.04	0.003
FEV1 (L)	2.57 ± 0.04	2.72 ± 0.02	2.72 ± 0.03	0.001
Age (years)	54.8 ± 0.7	55.8 ± 0.3	58.9 ± 0.5	<0.001
Males (%, N)	68 (28.7)	416 (47.3)	274 (57.3)	<0.001
Body mass index (kg/m^2)	24.6 ± 0.2	24.5 ± 0.1	24.3 ± 0.1	0.106
Education (elementary school/middle school/high school/university) (%)	22.4/22.0/31.5/24.1	27.6/15.1/33.1/24.3	35.6/18.5/28.6/17.3	<0.001
Smoking status (never/former/current smoker) (%)	70.3/13.4/16.4	55.6/23.8/20.6	48.4/31.8/19.8	<0.001
Current drinker (%, N)	40.5 (94)	55.8 (487)	56.4 (268)	<0.001
Physical activity (%, N)	41.4 (96)	42.2 (368)	47.0 (223)	0.186
SBP (mmHg)	124.2 ± 1.2	122.4 ± 0.5	124.1 ± 0.8	0.043
DBP (mmHg)	78.2 ± 0.7	78.2 ± 0.4	77.8 ± 0.5	0.233
TG (mg/dL) [2]	157.2 ± 7.6	152.5 ± 4.8	142.6 ± 3.8	0.024
TC (mg/dL)	195.0 ± 2.5	195.6 ± 1.2	192.7 ± 1.6	0.854
HDL-C (mg/dL)	47.9 ± 0.9	48.6 ± 0.4	48.6 ± 0.5	0.020
LDL-C (mg/dL)	117.7 ± 2.1	118.5 ± 1.1	115.8 ± 1.5	0.973
FBG (mg/dL) [2]	99.7 ± 1.2	103.0 ± 0.8	101.7 ± 0.8	0.681
HbA1c (%) [2]	5.83 ± 0.05	5.91 ± 0.03	5.90 ± 0.03	0.595
AST (IU/L) [2]	27.0 ± 3.1	23.6 ± 0.3	24.3 ± 0.5	0.899
ALT (IU/L) [2]	25.2 ± 3.5	23.3 ± 0.6	22.7 ± 0.6	0.772

FVC, forced vital capacity; FEV1, forced expiratory volume in 1 second; SBP, systolic blood pressure; DBP, diastolic blood pressure; TC, total cholesterol; TG, triglycerides; HDL-C, high-density lipoprotein cholesterol; LDL-C, low-density lipoprotein cholesterol; FBG, fasting blood glucose; HbA1c, glycated hemoglobin A1c; AST, aspartate aminotransferase; ALT, alanine aminotransferase. Values were expressed as means ± standard error for continuous variables and number of counts and percentage for categorical variables.[1] Statistical differences among genetic risk score were determined using general linear model for continuous variables after adjusting for age and sex and chi-squared test for categorical variables. [2] Tested after log-transformation.
